# Ineffective insurance in lower and middle income countries is an obstacle to universal health coverage

**DOI:** 10.7189/jogh.08.020402

**Published:** 2018-12

**Authors:** Abdulrahman M El-Sayed, Daniel Vail, Margaret E Kruk

**Affiliations:** 1 Department of Epidemiology, Mailman School of Public Health, Columbia University, New York, New York, USA; 2 Stanford University School of Medicine, Palo Alto, California, USA; 3 Department of Global Health & Population, TH Chan School of Public Health, Harvard University, Boston, Massachusetts, USA

## Abstract

**Background:**

Recent health policy efforts have sought to promote universal health coverage (UHC) as a means of providing affordable access to health services to populations. However, insurance schemes are heterogeneous, and some schemes may not provide necessary services to those covered. We explored the prevalence and determinants of ineffective insurance across 42 lower and middle income countries (LMICs) from the 2002-2004 World Health Survey.

**Methods:**

Respondents were defined as having ineffective health insurance if they reported being insured and: were forced to borrow or sell personal items to pay for health services; had an untreated chronic condition; or had recently delivered a child outside of a skilled health facility (women only).

**Results:**

Among the insured, 13% had ineffective insurance, which was most commonly due to having to borrow or sell to pay for health care. The likelihood of ineffective insurance was lowest in upper-middle income countries and higher in other lower-middle and low-income countries. Ineffective insurance also decreased with family wealth and was higher among rural residents.

**Conclusions:**

Our findings suggest that a high proportion of insurance in LMICs is ineffective, particularly among those who need it most, and that attention should be paid to effectiveness when defining health insurance in policy conversations about UHC.

Health insurance serves two primary functions for individuals. First, insurance secures financial access to health care for individuals both for preventive services and/or treatment and palliation in the setting of disease or injury [1]. Second, insurance evens the costs of those services, protecting against potentially devastating economic shocks that can occur as a result of care-seeking for illness [1]. For these reasons improving access to insurance coverage has recently become a goal of health policy efforts to improve health and well-being and reduce the financial burden of disease in low- and middle-income countries (LMICs).

These efforts have largely focused around the effort to achieve Universal Health Coverage (UHC) in LMICs, which has become an important centerpiece of global health policy [2-4]. A recent United Nations resolution, for example, “recognizes the responsibility of governments to urgently and significantly scale up efforts to accelerate the transition towards universal access to affordable and quality health-care services” [5]. Universal Health Coverage is one of the health goals in the new Sustainable Development Goals [6].

However, insurance plans are highly variable in the scope of benefit package, the magnitude of premiums, deductibles, and copayments, and the range of providers and health facilities participating in the network [7]. Health systems charged with providing covered services are highly heterogeneous as well. Previous studies have documented failures on the part of health insurance schemes to provide access to care or financial risk protection. These failures may come as a product of the quality of insurance itself, or the health care delivery context within which that insurance is operating [8]. For example, one study of public insurance plans in several Indian states demonstrated increases in catastrophic spending among beneficiaries as collective health expenditures increased [9]. Another study of intrapartum care in Ghana estimated that although delivery in a health facility was estimated at 68%, “effective coverage” of skilled attendance, that is coverage with services of acceptable quality, was a mere 18% [10].

Studies that focus on insurance coverage without paying attention to the effectiveness of that coverage may neglect important quality and financial gaps, which can undermine the intended outcomes of policy efforts. Understanding shortcomings in health system quality is particularly important given that the poor are disproportionately likely to be affected by coverage gaps [11]. To address this issue, we used data from the World Health Surveys of 42 LMICs to estimate the prevalence and determinants of ineffective insurance among respondents who reported having insurance coverage. We defined ineffective insurance as having health insurance but being unable to a) obtain treatment for diagnosed non-communicable diseases, b) delivering outside of a health facility (among women), or c) borrowing money or selling household assets to pay for health care services.

## METHODS

### Data

Data were collected by the World Health Organization as part of the 2002-2004 World Health Surveys (WHS). The WHS was conducted in 70 countries, representing each UN sub-region of the world, as well as countries from every income category defined by the World Bank (low, lower-middle, upper-middle, and high-income). Each survey provided country-specific sampling weights to allow for representative inference at the national-level. The WHS included questions on household characteristics as well as individual-level characteristics for the household’s primary respondent.

The initial WHS sample included data from 288 431 households in 70 countries. We restricted our sample to include respondents from low- and middle-income countries, based on the World Bank’s 2013 categorizations of country income. We chose to use 2013 categorizations of LMIC status to avoid including countries that were considered middle-income in 2003 but were rapidly transitioning into high-income countries (Croatia, Czech Republic, Estonia, Latvia, Slovakia, and Uruguay). In total, we excluded 27 high-income countries from our analysis (Australia, Austria, Belgium, Croatia, Czech Republic, Denmark, Estonia, Finland, France, Germany, Greece, Ireland, Israel, Italy, Latvia, Luxembourg, Netherlands, Norway, Portugal, Russia, Slovakia, Slovenia, Spain, Sweden, the United Arab Emirates, the United Kingdom, and Uruguay). Households from Guatemala were also excluded because the country survey did not provide sample weights. Additional households in the remaining 42 low- and middle-income countries (n = 240 943) were dropped from the sample due to: missing survey weights (n = 1596, 0.7%); missing information on insurance coverage (n = 40 191, 16.7%); or missing asset data necessary to construct wealth indices (n = 20 566, 8.5%), yielding a final analytic sample of n = 186 504, including 14 upper-middle income countries, 16 lower-middle income countries, and 12 low-income countries (**Table 1**).

**Table 1:** List of countries participating in the World Health Surveys, number of households included for analysis (n = 186 504), and the proportion of each country’s population participating in the country survey, categorized by 2013 World Bank income classifications

	Low income				Lower-middle income				Upper-middle income		
**Country**	n*	**pop %†**	**GDP (US$)‡**	**Country**	**n**	**pop %**	**GDP (US$)**	**Country**	**n**	**pop %**	**GDP (US$)**
Bangladesh	2622	3.50%	372	Côte d'Ivoire	2496	0.40%	812	Bosnia and Herzegovina	1005	0.10%	2148
Burkina Faso	4599	0.30%	332	Georgia	2692	0.10%	922	Brazil	450	4.70%	3040
Chad	4052	0.20%	294	Ghana	3346	0.50%	376	China	3915	32.90%	1274
Comoros	1647	0.00%	557	India	7340	26.80%	565	Dominican Republic	4738	0.20%	2345
Congo	1403	1.40%	1,039	Lao	4877	0.20%	360	Ecuador	1605	0.40%	2442
Ethiopia	4425	1.70%	120	Mauritania	2583	0.10%	433	Hungary	583	0.30%	8365
Kenya	4067	0.80%	440	Morocco	2113	0.80%	1663	Kazakhstan	4332	0.40%	2068
Malawi	5226	0.30%	198	Pakistan	4107	3.90%	546	Malaysia	5873	0.60%	4427
Mali	4147	0.30%	389	Paraguay	5221	0.20%	1159	Mauritius	3763	0.00%	4588
Myanmar	6032	1.10%	255	Philippines	9913	2.20%	1016	Mexico	38292	2.70%	6601
Nepal	305	0.70%	258	Senegal	998	0.30%	643	Namibia	3842	0.00%	2489
Zimbabwe	3620	0.30%	452	Sri Lanka	4751	0.50%	985	South Africa	1849	1.10%	3625
				Swaziland	1821	0.00%	1704	Tunisia	4880	0.30%	2790
				Ukraine	1080	1.20%	1049	Turkey	8303	1.70%	4595
				Vietnam	3677	2.10%	531				
				Zambia	3914	0.30%	450				

*n is number of households included for analysis by country.

†Pop % is the proportion of the total population of all countries included in the surveys as a proportion of that country’s population in 2003 based on data from CIA World Factbook.

‡GDP per capita is expressed in US dollars (USD) and is based on World Bank 2003 income data.

We intended to explore and document the degree, criteria, and predictors of ineffective insurance among households claiming to have insurance. Households self-reported the insurance status of the primary household respondent (“Is this person covered by any kind of health insurance plan?”). Respondents with an affirmative answer were considered insured. This insurance coverage was deemed ineffective if there was evidence that the insured individual was not receiving adequate health care or experienced financial duress from obtaining health care.

Respondents were considered ineffectively insured if they experienced one or more of the following criteria despite reporting having insurance: lack of treatment for a diagnosed chronic condition; failure to deliver a child in a health facility (women only); and the sale of household assets or borrowing money from someone other than a friend or family member in order to pay for health care in the past year. Respondents were considered to lack treatment for a chronic condition if they reported having been diagnosed with arthritis, angina, asthma, depression, schizophrenia/psychosis, or diabetes (the six non-communicable diseases for which data were available in the WHS) and answered “No” to the question “Have you ever been treated for [the disease]?” Female respondents were asked “Where did you give birth to [name of youngest child born in the last 5 years]?” and were considered to have given birth in a skilled health facility if they reported delivering in a hospital, maternity ward, or other health facility. Finally, respondents were asked “In the last 12 months, which of the following financial sources did your household use to pay for any health expenditures?” Those who reported selling household assets (such as furniture, animals, or jewelry) or borrowing money from someone other than a friend or family member, were considered to have ineffective insurance. These measures of health insurance efficacy are not intended to be comprehensive – it is perfectly feasible that a respondent to the WHS might have poor quality health insurance and yet still not be diagnosed with a chronic condition, not have a recent delivery, and not have had to sell household assets to pay for care. Instead, we use these variables as conservative indicators of insurance coverage that fails to accomplish the most basic goals of health insurance – smoothing the costs of health care and allowing policy holders to have access to care when necessary.

The following covariates were also considered during the course of our analysis, selected because of documented associations with health and health care utilization: age in years (categorical: 13-34, 35-65, 65+), sex, marital status (binary: married or cohabiting vs other), education (binary: any secondary education, no secondary education), urban residence (binary: urban, rural), country-specific wealth quintile (categorical: poorest, poor, middle, rich, richest 20%), country income category based on the World Bank’s 2013 classifications (categorical: low-income, lower-middle income, upper-middle income), and a dummy variable for each country to account for differences in national policies and health systems. Relative wealth indices were created within each country using principal components analysis of country-specific household asset questions; households were then divided into quintiles [12]. Fifteen to twenty questions were used in the construction of each index.

As this study uses publicly available secondary data from the World Health Organization, it was exempt from IRB review.

### Analysis

**Figure 1:**
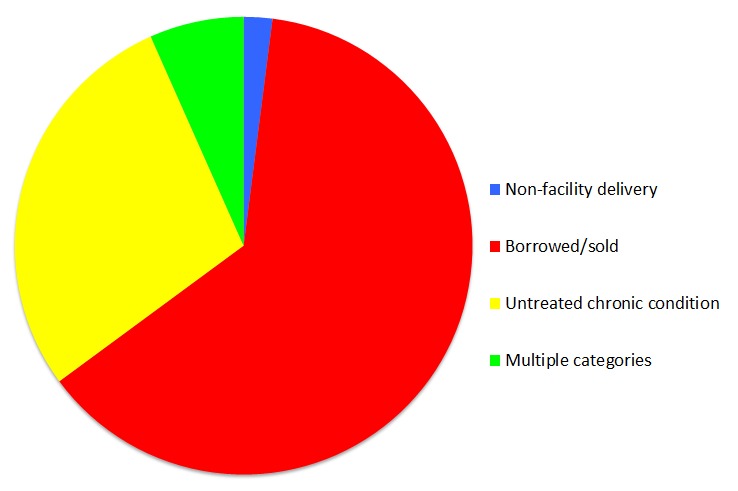
Categories of ineffective insurance among 7284 World Health Survey respondents who reported having insurance coverage, 2002-2004.

**Figure 2:**
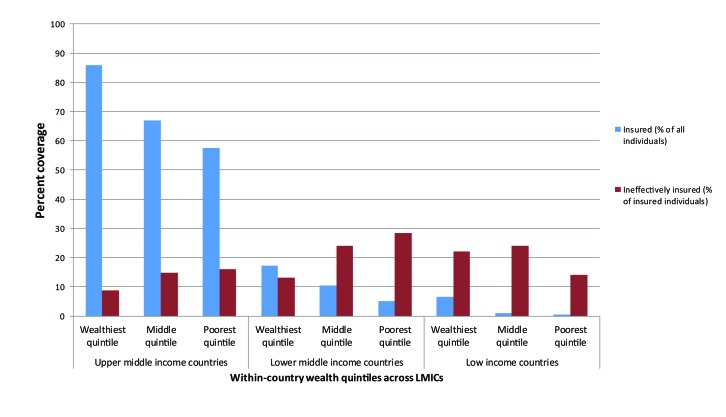
Total and ineffective insurance coverage of 186 504 World Health Survey respondents by within-country wealth quintiles across country income category, 2002-2004.

First, we calculated survey-weighted summary statistics to compare demographics across insurance status (**Table 2**), to show which indicators of ineffective insurance were most common (**Figure 1**), and to compare ineffective insurance prevalence by country income category (**Figure 2**). Second, we fit survey-weighted logistic regression of insurance status by demographic covariates (**Table 3**). In separate models, outcomes of interest included lack of insurance coverage (those respondents who did not claim to have insurance), and ineffective insurance, as well as each of the three indicators used in our definition of ineffective insurance (**Table 3**). Third, to demonstrate the joint impact of the covariates on likelihood of ineffective insurance we calculated the predicted probability of having ineffective insurance, conditional on age, gender, marital status, education, urban residency, and wealth, for two highly-contrasting theoretical respondents using coefficients from the logistic regression models in **Table 3** (**Table 4**), including an married woman between 13-34 years old without any secondary education in the poorest wealth quintile in a rural context and an unmarried man aged 65 years or older with secondary educated in the wealthiest quintile in an urban context.

**Table 2:** Demographics by insurance coverage and indicators of ineffective insurance among 181 238 World Health Survey respondents from 42 countries, 2002-2004*

	Overall (n = 186 504)	Insured (n = 51 207)†	Ineffectively insured (n = 7284)‡	Borrowed/ sold (n = 5614)¶	No treatment for chronic condition (n = 1847)§	Non-facility delivery (n = 383)‖
	**n (%)**	**n (%)**	**n (%)**	**n (%)**	**n (%)**	**n (%)**
**Overall**	186 504 (100.00)	51 207 (30.46)	7284 (12.82)	5614 (8.98)	1847 (4.40)	383 (0.36)
**Demographics:**						
Male	82 577 (49.64)	22 276 (52.74)	2872 (45.65)	2337 (44.32)	680 (51.52)	-
Age in years, mean (SD)	40.80 (0.18)	42.66 (0.43)	41.88 (0.82)	40.70 (0.94)	43.24 (1.37)	33.37 (1.43)
Married	116 585 (64.13)	28 679 (59.16)	4234 (62.35)	3016 (62.38)	1172 (60.39)	354 (80.60)
Secondary education	79 285 (47.39)	33 263 (69.53)	4365 (60.15)	3233 (57.12)	1151 (65.01)	218 (39.43)
Urban	89 496 (49.21)	35 573 (77.26)	4465 (70.96)	3287 (68.31)	1316 (79.94)	177 (41.80)
Wealth quintiles:**						
Highest	37 913 (23.25)	14 541 (33.28)	1393 (25.34)	927 (19.07)	498 (35.18)	55 (15.40)
High	37 915 (20.02)	11 581 (21.00)	1522 (16.33)	1108 (15.22)	438 (18.68)	77 (24.12)
Middle	37 217 (18.75)	10 557 (17.36)	1561 (22.01)	1238 (21.96)	355 (18.82)	94 (31.96)
Low	36 851 (18.66)	8181 (14.73)	1429 (18.03)	1162 (20.38)	319 (15.01)	73 (17.83)
Lowest	38 608 (19.33)	6347 (13.63)	1379 (18.29)	1179 (23.37)	237 (12.30)	84 (10.68)
**Country income categories:††**						
Upper middle	83 430 (36.74)	42 324 (84.53)	5266 (77.34)	4083 (74.78)	1361 (86.42)	136 (6.63)
Lower middle	60 929 (38.50)	7730 (13.52)	1760 (19.16)	1317 (22.23)	426 (10.54)	205 (68.45)
Lowest	42 145 (24.77)	1153 (1.94)	258 (3.49)	214 (2.99)	60 (3.04)	42 (24.92)

SD – standard deviation

*Number of respondents reported adjacent to survey-weighted percentage of total sample. All data are from the 2002-2004 World Health Survey, conducted by the World Health Organization.

†Includes all respondents who state that they are insured, regardless of the efficacy of that insurance.

**‡**Includes all respondents who state that they are insured, but who also reported experiencing one of the indicators of ineffective insurance delineated in the following three columns.

¶Includes all insured respondents who sold assets (for example, furniture, animals, or jewelry) or borrowed money from someone other than a friend or family member to pay for health expenses.

**§**Includes all insured respondents who were diagnosed with, but did not receive treatment for, one of the following six chronic conditions: arthritis, angina, asthma, depression, schizophrenia/psychosis, and diabetes.

**‖**Includes all insured female respondents who delivered a child in the past five years outside of a health facility.

**Within-country wealth indices were constructed by principle components analysis of a household asset index. Indices were divided into wealth quintiles within each country.

††Country income categories based on the World Bank’s 2013 categorization of lower and middle income countries.

**Table 3:** Adjusted odds ratios for insurance coverage, ineffective insurance coverage, and indicators of ineffective insurance, using survey-weighted logistic regression models: World Health Survey (2002-2004)*

			Indicators of ineffective insurance‡			
	**No insurance**	**Ineffective insurance†**		**Sold/borrowed**	**No treatment for chronic condition**	**Non-facility delivery**
	OR (95% CI)	OR (95% CI)		OR (95% CI)	OR (95% CI)	OR (95% CI)
**Demographics**	n = 166 781	n = 41 091		n = 41 209	n = 42 304	n = 30 680
**Age (in years, base = 65+ years):**						
13-34	1.37 (1.11, 1.69)	1.44 (0.74, 2.81)		1.85 (0.89, 3.83)	1.18 (0.41, 3.34)	n/a
35-65	1.07 (0.85, 1.34)	2.02 (1.11, 3.68)		2.12 (1.09, 4.13)	1.67 (0.71, 3.97)	n/a
Female	1.13 (0.98, 1.31)	1.29 (0.93, 1.80)		1.28 (0.92, 1.78)	1.04 (0.56, 1.95)	n/a
Married	0.84 (0.68, 1.03)	1.00 (0.70, 1.45)		1.05 (0.66, 1.69)	0.95 (0.61, 1.50)	1.66 (0.31, 8.99)
No secondary education	2.34 (2.03, 2.70)	1.06 (0.72, 1.56)		1.05 (0.73, 1.53)	1.04 (0.49, 2,21)	2.41 (1.48, 3.95)
Rural	1.16 (0.91, 1.48)	1.74 (1.21, 2.49)		1.63 (1.18, 2.26)	1.25 (0.60, 2.60)	2.04 (0.99, 4.18)
**Wealth quintile (base = highest):¶**						
Fourth	2.01 (1.64, 2.46)	1.02 (0.69, 1.52)		1.29 (0.84, 2.00)	0.89 (0.44, 1.78)	2.15 (1.13, 4.08)
Middle	2.76 (2.19, 3.47)	1.82 (1.06, 3.13)		2.42 (1.35, 4.33)	1.08 (0.48, 2.44)	4.83 (1.55, 15.11)
Second	4.04 (2.95, 5.53)	1.84 (1.13, 3.00)		2.82 (1.68, 4.71)	1.11 (0.42, 2.92)	3.08 (1.30, 7.30)
Lowest	7.14 (5.36, 9.52)	1.92 (1.23, 3.01)		3.59 (2.14, 6.02)	0.88 (0.35, 2.24)	3.39 (1.41, 8.16)
**Country income (base = upper-middle income):**						
Lower-middle income	240.09 (100.09, 575.96)	3.96 (2.06, 7.61)		4.70 (2.06, 10.68)	1.19 (0.41, 3.50)	5.93 (1.37, 25.68)
Low income	109.60 (79.65, 150.82)	9.85 (5.55, 17.48)		16.75 (8.01, 35.02)	2.13 (0.91, 4.99)	2.49 (0.49, 12.46)

OR – odds ration, CI – confidence interval

*All results reported in odds ratios. Models adjusted for age, gender, marital status, any secondary education, urban residency, within-country wealth quintile, and home country’s income status as defined by the World Bank in 2013. Errors were clustered at the country level. All data are from the 2002-2004 World Health Survey, conducted by the World Health Organization.

†Respondents were considered to have ineffective insurance if they did claim to have insurance coverage but had also: sold assets (for example, furniture, animals, or jewelry) or borrowed money from someone other than a friend or family member to pay for health expenses; not received treatment for one of six chronic conditions the survey asked about (angina, asthma, depression, arthritis, schizophrenia, or diabetes); or delivered a child in the past five years outside of a health facility. Only respondents who claimed to have health insurance were included in this model.

‡These models included only respondents with insurance coverage.

Within-country wealth indices were constructed by principle components analysis of a household asset index. Indices were divided into wealth quintiles within each country.

**Table 4:** Predicted probabilities of ineffective insurance for two theoretical respondents with insurance (using results from **Table 3**)*

Demographics	Person 1	Person 2
Age	13-34	65+
Gender	Female	Male
Marital status	Married	Not married
Education	No secondary	Secondary
Urban/rural	Rural	Urban
Wealth quintile†	Poorest	Wealthiest
**Predicted outcomes**	**Predicted probability (95% CI)**	
Any ineffective insurance‡	21.89 (12.34, 31.43)	4.05 (1.27, 6.83)
Borrow/sold¶	19.77 (9.88, 29.64)	1.58 (0.26, 2.91)
Untreated chronic condition§	3.72 (0.36, 7.08)	2.80 (0.00, 5.68)
Non-facility delivery‖	2.89 (1.05, 4.73)	n/a

CI – confidence interval

*Results are predicted probabilities based on adjusted logistic regression models summarized in **Table 2****.** Models adjusted for age, gender, marital status, any secondary education, urban residency, within-country wealth quintile, home country, and home country’s income status as defined by the World Bank in 2013. All data are from the 2002-2004 World Health Survey, conducted by the World Health Organization.

†Within-country wealth indices were constructed by principle components analysis of a household asset index. Indices were divided into wealth quintiles within each country.

‡Respondents were considered to have ineffective insurance if they had experienced any of the conditions delineated in the following three rows.

¶Respondents were considered to have ineffective insurance if they had sold assets (for example, furniture, animals, or jewelry) or borrowed money from someone other than a friend or family member to pay for health expenses

§Respondents were considered to have ineffective insurance if they had not received treatment for one of six chronic conditions the survey asked about: angina, asthma, depression, arthritis, schizophrenia, or diabetes.

‖Respondents were considered to have ineffective insurance if they had delivered a child in the past five years outside of a health facility.

All analyses used a two-stage weighting method. First, country-specific sampling weights provided within each survey were used to construct a nationally-representative sample for each country. Second, respondents in each country were weighted so that all countries contributed to the final analysis equally; respondents were weighted by the inverse proportion of their country’s total sample size relative to the total global sample.

**Figure 3:**
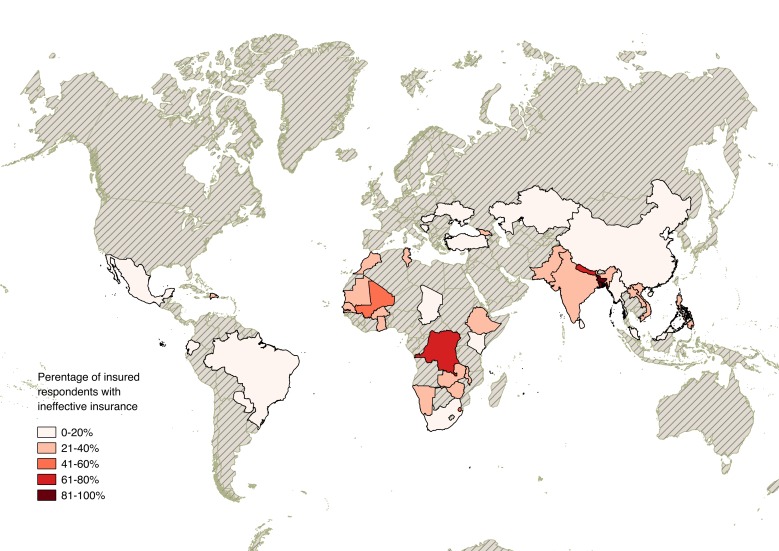
Ineffective insurance coverage by country using World Health Survey data (2002-2004).

Stata version 13 (StataCorp, College Station, TX, USA) was used for data analysis. QGIS was used to create the map of ineffective insurance coverage in **Figure 3**.

## RESULTS

**Table 2** shows demographic characteristics across our sample, the insured subsample, and the subsamples of insured respondents who reported borrowing or selling assets to pay for health care, not receiving treatment for a chronic condition, or failing to deliver a child in a health facility. Individuals with insurance coverage were, on average, older, more educated, more urban, more wealthy, and more likely to live in a higher income country than their uninsured counterparts. Insured individuals who had ineffective coverage were broadly dispersed across wealth quintiles. Respondents with insurance who reported borrowing or selling household assets or failing to receive treatment for a chronic condition were disproportionately more common in upper-middle income countries, while insured women who reported delivering outside of a skilled health facility were more common in lower-middle and lower-income countries.

**Figure 1** shows the distribution of criteria for ineffective insurance. Respondents were most likely to be ineffectively insured because they had borrowed or sold assets to pay for medical care (69%), with an untreated chronic condition being the next most prevalent indicator of ineffective insurance (34%). There was substantial overlap between respondents who borrowed to pay for care and respondents with an untreated chronic condition (6%), and relatively little overlap between respondents reporting non-facility delivery of a child and those reporting another criterion of ineffective insurance (<1%).

**Figure 2** shows the distribution of insurance and ineffective insurance by country income status across countries and household wealth within countries. Insurance coverage decreases with decreasing country income; coverage was 70% overall among upper-middle income countries, 11% among lower-middle income countries, and 2% among low-income countries. Similarly, ineffective insurance differed predictably by country income status. The prevalence of ineffective insurance was 12% overall among upper-middle income countries, 23% among lower-middle income countries, and 25% among low-income countries. Although the prevalence of insurance decreased with decreasing household wealth within each country income grouping, the prevalence of ineffective insurance increased with decreasing household wealth, save in low-income countries, where the prevalence was relatively even across all wealth quintiles.

**Table 3** shows adjusted odds ratios (ORs) for five insurance status outcomes (not having insurance, having ineffective insurance, and three measures of ineffective insurance) from survey-weighted multivariable logistic regressions among insured individuals. Relative household wealth within each country was associated with insurance coverage, as higher-income countries had higher prevalence of insurance and lower prevalence of ineffective insurance. At the individual level, the poorest 20% of respondents within each country had 1.92 (95% confidence interval (CI)  = 1.23-3.01) times’ higher odds of ineffective insurance than their wealthiest counterparts. Younger respondents were significantly more likely to be uninsured, and, if they were insured, to have ineffective insurance than counterparts aged 65 years or older. Rural respondents were 1.74 times more likely to have ineffective insurance than their urban counterparts (95% CI = 1.21-2.49). Older age, rural residence, lower wealth, and lower country income were associated with higher likelihood of selling or borrowing assets to pay for care. No secondary education, lower wealth and lower country income were associated with delivery outside of a skilled facility.

**Table 4** shows predicted probabilities from the regression model described in **Table 3** for two hypothetical insured individuals, an uneducated rural married woman aged 13-34 years in the lowest wealth quintile within her country, and a wealthy unmarried urban male older than 65 with secondary education. The former’s probability of ineffective insurance was 22%, while the latter’s was 4%.

**Figure 3** shows countries included in our final sample, shaded according to the proportion of insured respondents in each country with ineffective insurance. Insurance was least common in the same regions where insurance was most likely to be ineffective: Southern, Eastern, and Western Africa, as well as South Asia.

## DISCUSSION

Our study of insurance among residents of 42 LMICs from the World Health Surveys yielded several important findings: First, nearly one in seven respondents who reported having insurance coverage did not have effective insurance, largely as a function of having to borrow or sell household goods to pay for health care services. Second, ineffective insurance was most common in the countries and households where insurance was least common, that is in the poorest households in the lowest income countries. Third, the poor and undereducated were most likely to have ineffective insurance. Taken together, our findings suggest that policies aiming to improve access to insurance as a means of promoting health and smoothing the costs of health care in LMICs should pay particular attention to the design of insurance, in particular its extent of financial protection and scope of the benefit package, as a substantial proportion of those with coverage may not, in fact, reap the health and financial protection benefit of insurance under current design – particularly the poor and underserved.

Our study contributes to a growing literature about effective insurance coverage. Acharya and colleagues reviewed the literature about the influence of insurance schemes among workers in the informal sector in LMICs [13]. They found that, in general, insurance schemes in these contexts were not associated with improvements in health care utilization, protection from financial consequences of illness, or improvements in health [13]. For example, Lei and Lin demonstrated no difference in health care utilization nor in out-of-pocket spending in the case of health shocks (ie, abrupt exogenous changes in health status) analyzing China’s rural cooperative medical scheme [14]. Where improvements were observed, they were usually relegated to the wealthier beneficiaries. However, there were notable exceptions in specific contexts. For example, Miller and colleagues analyzed the influence of the *Regimen Subsidiado* insurance scheme in Colombia, demonstrating increased utilization of preventive health services and blunted financial consequences of illness [15]. The literature therefore paints a complex picture of the influence of insurance on health care utilization and health expenditures in the setting of health shocks, likely related to insurance design and local health system effectiveness.

Our findings extend this literature in three principal ways. First, our work frames insurance relative to its teleological foci of improving access to health care services when necessary and protecting against the financial consequences of poor health. Second, we leverage a global data set to estimate the prevalence of insurance that does not meet these ends across 42 LMICs. Third, we considered the sociodemographic predictors of ineffective insurance to understand differences in the burden of ineffective insurance across individuals, households, and contexts.

Our findings demonstrated that, in general, the likelihood of insurance coverage was inversely related to the likelihood that that insurance was ineffective both by country-level income as well as household level wealth. Furthermore, we found that measures of low socioeconomic position, such as wealth in the lowest quintile and no secondary education predicted lower likelihood of any insurance, and higher likelihood of ineffective insurance. This suggests that insurance is most likely to be both lacking and ineffective among those who likely need it most as a function of their likelihood of morbidity [16] and their vulnerability to financial catastrophe due to health care use.

Our findings also demonstrate that the most common category of ineffective of insurance was incapacity to protect against the financial consequences of health-related shocks, as nearly 1-in-10 of those with insurance were forced to borrow or sell household items to pay for health care services. As those in the poorest quintile were nearly 4 times more likely as their wealthiest counterparts to borrow or sell to pay for services, and those in low-income countries were nearly 17 times more likely to do so than their counterparts in upper-middle income countries, our findings suggest that the financial consequences of ineffective insurance were disproportionately borne by the poor. Additionally, we suspect that our analysis underestimates the true prevalence of insurance that fails to protect against financial shocks, as respondents who forgo care entirely in the setting of unaffordable health costs will not end up selling household assets, and will therefore not be identified as having ineffective insurance.

Importantly, there are two principal mechanisms by which insurance may become ineffective. First, insurance simply may not operate to provide care or smoothen health care costs as a function of limited benefits, high deductibles, caps on reimbursements, or outright financial mismanagement. Second, and perhaps more insidiously, insurance is not sufficient to surmount the weaknesses of health systems within which individuals may be insured. Insurance schemes frequently cover a limited set of services and cannot guarantee the availability of health care providers, medications, or facilities, let alone their consistency, competence, or quality. For example, in low-income countries with high home-delivery rates, insurance status may not be the primary barrier to safe childbirth. It is plausible that a large proportion of ineffective insurance, particular in low-income, rural contexts, may thus fail to produce better health outcomes. Although this distinction was beyond the scope of the present analysis, it has important implications for understanding the potential and limitations of insurance as a health policy tool to improve health access and minimize the financial consequences of illness. Further analyses of this data set should explore the ways in which health outcomes differ between the ineffectively insured and the completely uninsured.

Our findings should be considered within the context of several important limitations. First, there are several limitations arising from the nature of our data, which was cross-sectional, international, and limited in scope. As a cross-sectional study, this work could not establish temporality between nominal insurance and the various criteria for which insurance was deemed ineffective; for example, as we don’t have the start date of insurance coverage some individuals in our sample may have obtained insurance coverage only after having borrowed or sold to pay for health care services. Similarly, a principal strength of our data are the international scope of the work, which also imposes limitations: the nature of insurance (eg, reimbursement levels, benefit package, and caps) may differ substantially by country, and generalizing may obfuscate these important differences despite inclusion of country indicator variables. Furthermore, we lacked data regarding the severity of non-communicable disease and/or the degree to which households were forced to borrow or sell household items to pay for health care services. Therefore, our data may obfuscate important differences in the degree and/or consequences of ineffective insurance. Our estimates of ineffective insurance are likely an underestimate. We chose relatively few, relatively strict criteria for ineffectiveness and if these were extended, for example to having received no diagnosis in the presence of disease symptoms, the prevalence would be higher. Additionally, information on insurance status was missing for approximately 16% of respondents in our sample. Finally, the WHS was collected in 2002-2004. There have important changes in the health care ecosystem in LMICs since then, including several important initiatives such as the World Health Organization’s Global Health Action Plan, 2013-2020. Despite this, the WHS remain the most comprehensive and recent global health surveys available. They therefore continue to yield insight into health service access in LMICs.

Despite these limitations, our findings have important implications for future research and policy. With respect to future research, understanding the risk, consequences, and predictors of ineffective insurance across various insurance schemes remains an important area of inquiry as intergovernmental organizations and governments continue to debate the merits of various schemes. Second, our work did not differentiate between the various mechanisms of ineffective insurance – those that occur as a function of insurance itself, and those that occur as a function of the health systems within which beneficiaries may be using that insurance. Understanding these mechanisms should take high priority in future work. Third, we did not here study the health consequences of ineffective insurance, which presents a fertile space for future research.

Furthermore, the policy implications of this work are clear. Not all insurance is effective in providing the key ends of promoting access to health care and financial protection. We estimated that in LMICs only 30% of people have insurance, and nearly 1-in-7 people with insurance have ineffective insurance. This should lead policymakers working on UHC to shift focus from extending nominal insurance to the greatest number to designing robust insurance schemes accompanied by commensurate investments in the scope and quality of covered health services.[17-19]. Therefore, as UHC continues to occupy a privileged mantle in health policy conversations, particular attention ought be paid to effective insurance coverage. This is particularly urgent if the promise of UHC is to be realized among society’s most vulnerable – the poor in low-income countries.
